# The Effect of Oral Mucosal Mesenchymal Stem Cells on Pathological and Long-Term Outcomes in Experimental Traumatic Brain Injury

**DOI:** 10.1155/2022/4065118

**Published:** 2022-04-26

**Authors:** Fatemeh Dehghanian, Zahra Soltani, Alireza Farsinejad, Mohammad Khaksari, Elham Jafari, Ali Darakhshani, Nazanin Sabet, Hamideh Bashiri

**Affiliations:** ^1^Neuroscience Research Center, Institute of Neuropharmacology, Kerman University of Medical Sciences, Kerman, Iran; ^2^Bam University of Medical Sciences, Bam, Kerman, Iran; ^3^Endocrinology and Metabolism Research Center, Institute of Basic and Clinical Physiology Sciences, Kerman University of Medical Sciences, Kerman, Iran; ^4^Research Center of Tropical and Infectious Diseases, Kerman University of Medical Sciences, Kerman, Iran; ^5^Physiology and Pharmacology Department, Afzalipour Faculty of Medicine, Kerman University of Medical Sciences, Kerman, Iran; ^6^Department of Hematology and Medical Laboratory Sciences, Faculty of Allied Medicine, Kerman University of Medical Sciences, Kerman, Iran; ^7^Physiology Research Center, Institute of Neuropharmacology, Kerman University of Medical Sciences, Kerman, Iran; ^8^Pathology and Stem Cell Research Center, Kerman University of Medical Sciences, Kerman, Iran

## Abstract

**Background:**

Neuroprotective effects of stem cells have been shown in some neurologic diseases. In this study, the effect of oral mucosal mesenchymal stem cells (OMSCs) on traumatic brain injury (TBI) was evaluated in long term.

**Materials and Methods:**

TBI was induced by Marmarou's method. The number of 2 × 10^6^ OMSCs was intravenously injected 1 and 24 h after the injury. Brain edema and pathological outcome were assessed at 24 h and 21 days after the injury. Besides, long-term neurological, motor, and cognitive outcomes were evaluated at days 3, 7, 14, and 21 after the injury.

**Results:**

OMSCs administration could significantly inhibit microglia proliferation, and reduce brain edema and neuronal damage, at 24 h and 21 days after the injury. Neurological function improvement was observed in the times evaluated in OMSCs group. Cognitive and motor function dysfunction and anxiety-like behavior were prevented especially at 14 and 21 days after the injury in the treatment group.

**Conclusion:**

According to the results of this study, OMSCs administration after TBI reduced brain edema and neuronal damage, improved neurologic outcome, and prevented memory and motor impairments and anxiety-like behavior in long term.

## 1. Introduction

Traumatic brain injury (TBI) is one of the major causes of death and disability worldwide [1, 2]. Despite the effective therapeutic options available, TBI treatment remains a challenge for scientists and physicians [3]. TBI commonly results in primary and secondary injury. Primary injury is caused by mechanical events, followed by a cascade of pathological and biochemical changes leading to secondary injury and neuronal death [4]. Increased reactive oxygen species and energy consumption [4], damaged blood-brain barrier [5], activated inflammation [6], progressive neuronal destruction [7], apoptosis, and release of excitatory amino acids [8] are considered as mechanisms of secondary injury in TBI. These mechanisms lead to brain edema and increased intracranial pressure (ICP), which followed by neurological disorders [9] and cognitive impairments regardless of the severity of the injury [10]. Brain edema is a major cause of mortality and neurological disabilities caused by TBI [11].

Despite extensive investigations [12–14], successful treatment for TBI has not yet been reported [3]. Stem cells (SCs) have currently been attracting a lot of interest in neuroscience research because of their important role in regenerative medicine [15]. Studies over the past decade have provided great support for the use of different stem cells in the treatment of neurological deficits such as TBI and stroke [16]. Among postnatal adult stem cells, mesenchymal stem cells (MSCs) with pluripotent property have of particular importance [8]. In addition to bone marrow, these cells have recently been isolated from other tissues including bone, primary teeth, placenta, and oral mucosa [17] with properties similar to bone marrow MSCs [18]. Also, they can differentiate into nonmesenchymal cells such as neural tissue [19]. These cells are in the focus of attention of regenerative medical studies due to their easy access, resource abundance, and high power of differentiation [16].

Among MSCs, oral mucosal mesenchymal stem cells (OMSCs) have been described as cells originating from the neural crest [17]. These cells are important for a wide range of scientific studies because of significant healing power, high SC population and availability due to being clonogenic cells [20], ability to differentiate into neural cells, fast proliferation, and stable morphology [17]. OMSCs can differentiate into astrocyte-like cells and are capable of producing neuroprotective factors such as brain-derived neurotrophic factor (BDNF), vascular endothelial growth factor (VEGF), glia-derived neuroprotective factor (GDNF), and insulin-like growth factor-1 (IGF-1). Considering the neuroprotective effects of these cells in vitro [21] and in vivo [22], their study on the treatment of TBI has been proposed.

Administration of other types of stem cells including neural stem cells (NSCs) is also effective in treating TBI by enhancing cell proliferation and neurogenesis and improving function [23]. In another study, bone marrow-derived MSCs decreased the permeability of the blood-brain barrier, neuronal inflammation, and microglial accumulation in brain tissue [24]. Prompt administration of MSCs after injury intracranially enhances survival, proliferation, and differentiation of NSCs and expression of NSCs-stimulating cytokines [25]. Consumption of human adipose-derived stem cells (hADSCs) improves cognitive deficits by reducing cortical lesion size and inhibiting cell death in the hippocampus [26].

Based on the absence of successful and effective treatment for TBI patients [8], the existence of evidence shows neuroprotective effects of MSCs on neurodegenerative diseases including TBI [2] and also the benefits of using OMSCs in nervous diseases [22]; the present study set out to assess the effect of OMSCs on TBI. In this investigation, the effect of OMSCs administration on brain edema, anxiety-like behavior, and pathological, neurological, cognitive, and motor outcomes induced by moderate diffuse TBI in male rats in long term were evaluated. The authors confirm that a preprint of the article has previously been published in Research Square [27] .

## 2. Materials and Methods

### 2.1. Animals

This experimental interventional study was approved by the Ethical Committee of Kerman University of Medical Sciences with Code of Ethics IR.KMU.REC.1397.210. Animal care and behavioral tests were conducted in accordance with the standard ethical guidelines, and all efforts were made to minimize animal suffering. The experiments were performed on adult male Wistar rats weighing 200-220 g and bred in Kerman University of Medical Sciences. Animals were housed in a temperature-controlled room (25^°C^) for 12 h-light/dark cycle, with free access to water and food.

### 2.2. Experimental Procedure

Forty-eight animals were randomly divided into four groups as follows:

(*i*) Sham: Rats received all necessary procedures to cause diffuse TBI except falling weight on their head

(*ii*) TBI: Rats received a trauma by a 300 g weight on their head

(*iii*) Vehicle (Veh): Rats intravenously received phosphate-buffered saline (PBS) in a volume of 100 *μ*L at 1 and 24 h after TBI [28]

(*iv*) Stem cell (SC): Rats intravenously received 2 × 10^6^ OMSCs [29] in a volume of 100 *μ*L at 1 and 24 h after TBI

### 2.3. Isolation of OMSCs

OMSCs were obtained from rat's oral mucosa biopsies. The extracted tissue was washed with sterile PBS and enzymatically digested with 2% type I collagenase under shaking conditions. Collagenase activity was neutralized with an equal volume of low glucose Dulbecco's modified Eagle's medium (L-DMEM) containing 10% fetal bovine serum (FBS). To isolate OMSCs, the filtered cells were centrifuged at 2000 rpm/min for 10 min. The cell pellet was filtered by a 70-mm pore-size filter. The cells were cultured in L-DMEM supplemented PBS and 100 IU/mL penicillin +100 *μ*g/mL streptomycin during two weeks. The cells incubated at 36.5°C, 5% CO2. The culture medium was changed every 72 h and the cells were passaged by trypsin enzyme [25]. In a pilot study, the number of 2 × 10^6^ [29], 4 × 10^6^ [30], and 8 × 10^6^ OMSCs in 100 *μ*L PBS was intravenously injected to rats (*n* = 5 in each group) at 1 and 24 h after TBI. Thereafter, their effects on brain edema and histopathologic outcome were evaluated at 24 and 48 h after TBI. Effective number of cells was determined when indices were compared to the control group.

### 2.4. Model of Diffuse Traumatic Brain Injury

Rats were anesthetized with the injection of ketamine (50 mg/kg) plus xylazine (10 mg/kg) intraperitoneally, and then, intubation of all animals was performed. The method was basically the same as described earlier [13]. A stainless steel plate 10 mm in diameter and 3 mm in thickness was attached to the skull bone between bergma and lambda. In all groups except the sham, a 300 g weight was dropped from a 2 m height onto the plate on the animal's head, causing diffuse TBI. Immediately thereafter, the rats were connected to a respiratory pump if it was needed. After restoration of spontaneous breathing, intratracheal tube was removed, and the animals were placed in an individual cage following recovery of the surgery.

### 2.5. Evaluation of Brain Edema

The brain edema was evaluated by measuring brain water content at 24 h and 21days after TBI. Animals were anesthetized, the brains were removed, and brain samples were located in pre-weighed vials and weighed (wet weight). The lids were lifted, and the vials were placed in an incubator at 100°C for 48 h, and afterward reweighed (dry weight). The percentage of water in the brain of each animal was calculated as follows: (100 × [(wet weight–dry weight)/wet weight]) [31].

### 2.6. Evaluation of Neurological Severity Score (NSS)

NSS was assessed on 3rd, 7th, 14th, and 21st days after TBI by a blind trained investigator to the experimental groups. NSS is an intricate behavioral test including motor, sensory, balance, and reflex tests. Scoring range is 0–18, in which higher scores reflect a greater extent of the injury. The scores of 0 and 18 indicate normal performance and maximal impairment, respectively. The scores of 1–6, 7–12, and 13–18 indicate mild, moderate, and severe injury, respectively (Table 1) [32].

### 2.7. Behavioral Assessment Tests

In the present study, tests of elevated plus maze (EPM), open field (OFT), and Morris water maze (MWM) were done to assess anxiety-like behaviors, locomotor activity, and spatial learning and memory, respectively, by an investigator who was blind to the study groups. Behavioral tests were performed at days 3, 7, 14, and 21 after TBI. All sessions of behavioral tests were video-recorded by cameras which were hung from the ceiling (2.5 m high) and located directly above the center of the mazes. These cameras were connected to computers in a neighboring room for saving the rat's behavior. All behavioral indices were recorded by a video tracking system software (Borje Sanat, Iran). To avoid the effect of circadian rhythm on animal's behavior, the tests were performed at a determined time of day in a quiet environment.

### 2.8. EPM Evaluation

An EPM apparatus was made of wood and consisted of two closed and two open arms with the equal size (50 × 10 cm). The close and open arms were enclosed by 40-cm-high and 0.5-cm-high walls, respectively. The four arms were linked by a central platform (10 × 10 cm). The apparatus was elevated 50 cm above the floor. The experiments were performed in a room lit by a 60-W light bulb located above the center of the EPM. The animals were placed in a room for acclimation 1 h before behavioral testing without observing the apparatus. Each rat was placed in the center of the EPM facing an opened arm and allowed 5 min of exploration. Entry was defined as four paws in the arms. The number of entries into close and open arms and the total time spent in the closed and open arms were measured. As the anxiety indices, the percentage of open arm time (%OAT: the ratio of times spent in the open arms to total times spent in any arms×100) and open arm entries (%OAE: the ratio of entries into open arms to total entries×100) were calculated. Moreover, total arm entries were evaluated as a relative pure parameter of locomotor activity [33].

### 2.9. OFT Evaluation

An OFT apparatus was constructed of plexiglass and consisted of a square arena (90 × 90 × 30 cm), which was divided by lines into 16 equal squares. Each rat was located in the central zone and allowed 5 min of exploration. All experiments were performed in a dimly illuminated testing room. The velocity (cm/s) of animals and total distance moved (cm) were measured [34].

### 2.10. MWM Evaluation

The Morris water maze is an authentic apparatus to assess spatial learning and memory in laboratory animals. This task is a circular tank, 150 cm in diameter and 60 cm in depth, filled with water (23-25°C). The animals ran away from water onto an invisible platform (10 cm wide, 35 cm high), which located 1.5 cm beneath the water level. The MWM was surrounded by different visual cues on the testing room walls, and their place remained unchanged throughout the test period. The maze was divided into four quadrants, and the animals were placed in one of the four equal quadrants, randomly. The parameters such as the total time spent in the target quadrant and the number of entries to the target quadrant were measured. The training session included three blocks on three consecutive days, and each block comprised of four consecutive trials. On each trial, rats were randomly dropped into the maze from a defined point of each quadrant and were allowed to swim for 60 seconds to find the hidden platform. After the detection of the platform, the animal remained there for 20–30 s and then was caged for 20–30 s before the next trial. The retention of spatial memory was assessed 24 h after training trials by removing the platform in a 60 s probe trial [35].

### 2.11. Histopathology Evaluation

The histopathological outcome was evaluated on the first and 21st day after TBI. Briefly, the brain tissue was washed with 0.9% cold saline, fixed in 10% formalin and, after tissue processing, embedded in paraffin, and sectioned into 5 *μ*m using a microtom (Leica RM 2156, Germany). Slides were prepared and stained with hematoxylin and eosin (H&E). Neuronal damage (pink to red ischemic neurons with perineuronal vacuolation) and microglia proliferation in the brain tissue were assessed under a microscope (Olympus CX33, Japan) by a pathologist who was blind to the experimental groups. The semiquantitative scores reflected the approximate number of damaged neurons manifesting ischemic changes in the perilesional cortex in the groups examined (1 = <5%, 2 =6% -20%, 3 =21%-50%, 4 =51%-75%, and 5 = 76%-100%) [36]. The microglia proliferation was reported as +1, +2, +3, and+4 [14].

### 2.12. Statistical Analysis

Results were reported as mean ± SEM. Normality of data was checked using the Shapiro-Wilk test. Data with normal distribution was analyzed using two-way repeated measures ANOVA to compare the mean data between groups at different times, except for variables of brain water content and histopathologic indices. Only the results of the comparison between groups in the analysis were reported considering the goal of the study. The results of brain water content were analyzed using one-way ANOVA, while histopathologic indices were analyzed using Kruskal-Wallis. In all statistical comparisons, *p* values less than 0.05 were considered as the criterion for statistical significance. Data analyses were done using the SPSS software package version 20 (SPSS Inc., Chicago, IL, USA).

## 3. Results

### 3.1. OMSC Administration Prevented Increasing the Brain Water Content after TBI

**Figure 1:**
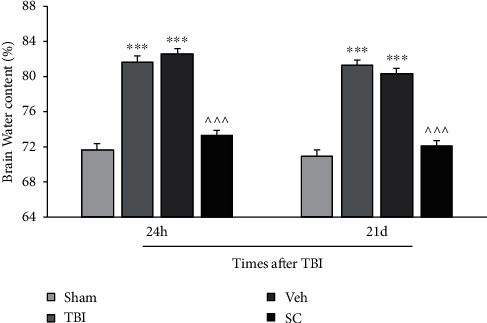
Comparison of brain water content (%) in study groups at 24 h and 21 days after injury (*n* = 6). Each bar represents mean ± SEM. ∗∗∗*P* < 0.001 compared with the sham group. ^∧∧∧^*P* < 0.001 compared with the vehicle group. TBI: traumatic brain injury; Veh: vehicle; and SC: stem cell.

The comparison of brain water content between the experimental groups was performed at 24 h and 21 days after trauma. As shown in Figure 1, brain water content increases after brain injury compared to the sham group at both times (*P* < 0.001). The administration of SC lessened the brain water content compared to the vehicle group at 24 h and 21 days after injury (*P* < 0.001). At 24 h and 21 days after injury, the brain water content in the group receiving SC did not differ from the sham group.

### 3.2. OMSC Administration Ameliorated Long-Term Neurological Outcome

**Figure 2:**
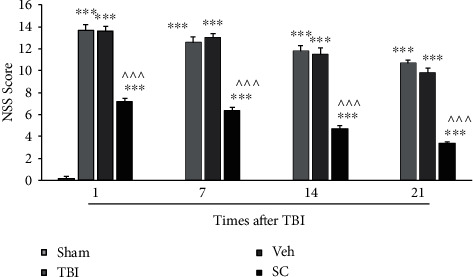
Comparison of neurological severity score (NSS) in study groups at 3, 7, 14, and 21 days after injury (*n* = 6). Each bar represents mean ± SEM. ∗∗∗*P* < 0.001 compared with the sham group. ^∧∧∧^*P* < 0.001 compared with the vehicle group. TBI: traumatic brain injury; Veh: vehicle; and SC: stem cell.

The analysis showed that there was an interaction between group and time for NSS variable (*P* < 0.001), and the main effect of score for both time and group was *P* < 0.001. At all times after injury, a significant increase in NSS was observed in the TBI and vehicle groups compared with the sham group (*P* < 0.001). NSS was higher in SC group compared to the sham group (*P* < 0.001). On the other hand, SC group was able to significantly reduce the amount of NSS compared to the vehicle group (*P* < 0.001) (Figure 2).

### 3.3. OMSC Prevented Anxiety-Like Behaviors after TBI

**Figure 3:**
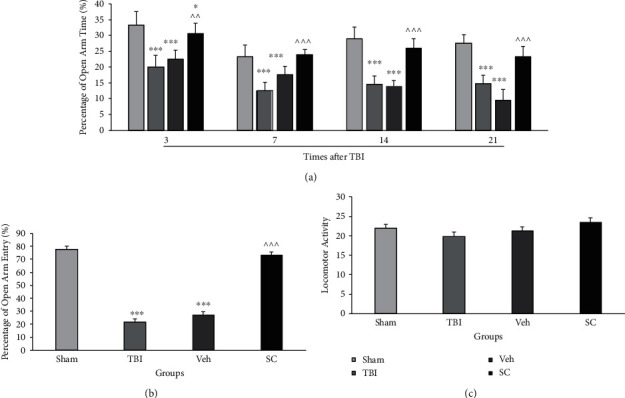
Comparison of open arm time (%) (a), open arm entry (%) (b), and locomotor activity (c) in the elevated plus maze (EPM) in study groups at 3, 7, 14, and 21 days after injury (*n* = 6). Each bar represents mean ± SEM. ∗*P* < 0.05, ∗∗∗*P* < 0.001 compared with sham group. ^∧∧^*P* < 0.01, ^∧∧∧^*P* < 0.001 compared with vehicle group. TBI: traumatic brain injury; Veh: vehicle; and SC: stem cell.

In this study, %OAT, %OAE, and locomotor activity as indices of anxiety-like behaviors in the EPM were assessed on days 3, 7, 14, and 21 after injury. The analysis showed that there was an interaction between group and time for %OAT variable (*P* < 0.001). The main effect of %OAT for both time and group was *P* < 0.001.  Figure 3(a) illustrates the percentage of time spent in the open arm of the EPM in study groups. %OAT decreased in the TBI and vehicle groups compared with the sham group at all days after injury (*P* < 0.001). The %OAT was significantly increased in the SC group compared to the vehicle group at days 3 (*P* < 0.01), 7, 14, and 21 after injury (*P* < 0.001).

The analysis showed that there was no interaction between group and time for %OAE (*P* = 0.52). Main effect of %OAE for time and group was *P* = 0.68 and *P* < 0.001, respectively. A comparison of the %OAE in study groups is shown in Figure 3(b). This variable was lower in the TBI and vehicle groups than in the sham group (*P* < 0.001). The %OAE increased in the SC group compared to the vehicle group (*P* < 0.001).

There was no interaction between group and time for locomotor activity (*P* = 0.57), and the main effect of motor activity for time and group was *P* < 0.001 and *P* = 0.22, respectively. A comparison of the locomotor activity in sham, TBI, vehicle, and SC groups is shown in Figure 3(c). No significant difference was seen among groups.

### 3.4. OMSC Administration Reversed the TBI-Induced Velocity Decline in the OFT

**Figure 4:**
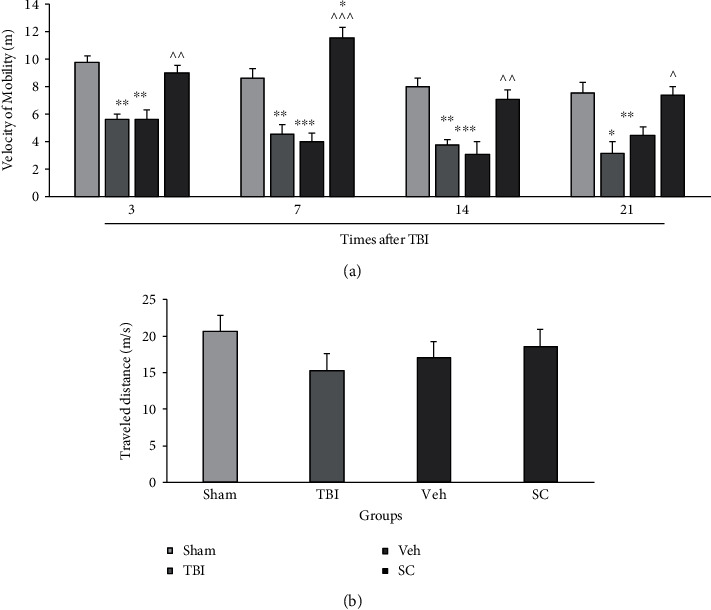
Comparison of velocity in the open field test (OFT) (a) and traveled distance (b) in study groups at 3, 7, 14, and 21 days after injury (*n* = 6). Each bar represents mean ± SEM. ∗*P* < 0.05, ∗∗*P* < 0.01, ∗∗∗*P* < 0.001 compared with sham group. ^∧^*P* < 0.05, ^∧∧^*P* < 0.01, ^∧∧∧^*P* < 0.001 compared with vehicle group. TBI: traumatic brain injury; Veh: vehicle; and SC: stem cell.

The velocity in the OFT was evaluated as the index of motor outcome. The analysis showed that there was an interaction between group and time for velocity variable in the OFT (*P* < 0.001) and the main effect of velocity for both time and group was *P* < 0.001.  Figure 4(a) shows the comparison of velocity in study groups at 3, 7, 14, and 21 days after injury. Velocity decreased in TBI and vehicle groups compared with sham group at days of 3 (*P* < 0.01), 7 (*P* < 0.01, *P* < 0.001), 14 (*P* < 0.01, *P* < 0.001), and 21 (*P* < 0.01, *P* < 0.05), respectively. The velocity increased in the SC group compared to the vehicle group at 3 (*P* < 0.05), 7 (*P* < 0.001), 14 (*P* < 0.01), and 21 (*P* < 0.05) days after injury.

The analysis showed that there was no interaction between group and time for traveled distance (*P* = 0.53), and the main effect of traveled distance for time and group was *P* < 0.01 and *P* = 0.42, respectively. A comparison of the traveled distance in sham, TBI, vehicle, and SC groups is shown in Figure 4(b). No significant difference was seen between groups.

### 3.5. OMSC Partly Improved Spatial Memory after TBI

**Figure 5:**
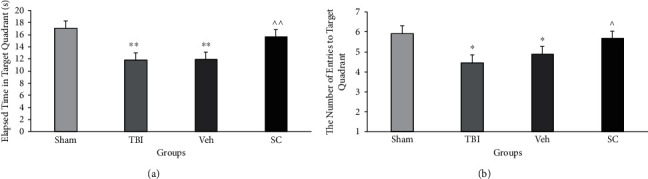
Comparison of time spent in the target quadrant during probe trial in Morris water maze (MWM) (a) and the number of entries to target quadrant during probe trial (b) in study groups at 3, 7, 14, and 21 days after injury (*n* = 6). Each bar represents mean ± SEM. ∗*P* < 0.05, ∗∗*P* < 0.01 compared with sham group. ^∧^*P* < 0.05 compared with vehicle group. TBI: traumatic brain injury; Veh: vehicle; and SC: stem cell.

Spatial memory was assessed using the time spent in the target quadrant and the number of entries to the target quadrant during probe trial in the MWM (Figure 5). No significant difference for distance traveled in the target quadrant was found (Data not shown).

The analysis showed that there was no interaction between group and time for the target quadrant during probe trial (*P* = 0.94). Main effect of time spent in the target quadrant for time and group was *P* = 0.85 and *P* = 0.002, respectively. The time spent in the target quadrant during probe trial in study groups is illustrated in Figure 5(a). This time decreased in the TBI and vehicle groups compared to the sham group (*P* < 0.01). This parameter increased in the SC group compared to the vehicle group (*P* < 0.01).

The analysis showed that there was no interaction between group and time for the number of entries to the target quadrant (*P* = 0.15). The main effect of the number of entries to the target quadrant for the time and group was *P* = 0.34 and *P* = 0.002, respectively.  Figure 5(b) shows the number of entries to the target quadrant during probe trial in study groups. This variable decreased in TBI and vehicle groups compared to the sham group (*P* < 0.05). This parameter increased in the SC group compared to the vehicle group (*P* < 0.05).

### 3.6. Histopathological Results

**Figure 6:**
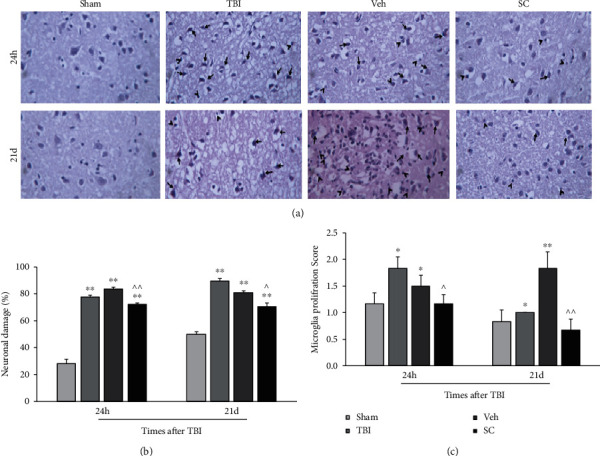
Histopathological images (hematoxylin & eosin, 400×) in study groups, microglia proliferation (arrow tip) and neuronal damage (arrow) (a). Comparison of neuronal damage score (b) and microglia proliferation score (c) in study groups at 24 h and 21 days after injury (*n* = 6). Each bar represents mean ± SEM. ∗*P* < 0.05, ∗∗*P* < 0.01 compared with the sham group. ^∧^*P* < 0.05, ^∧∧^*P* < 0.01 compared with vehicle group. TBI: traumatic brain injury; Veh: vehicle; and SC: stem cell.

To evaluate the pathologic outcome, parameters including neuronal damage and microglia proliferation in the brain tissue were evaluated (Figure 6). Histopathological images (hematoxylin & eosin, 400×) in sham, vehicle, TBI, and SC groups are shown in Figure 6(a).

At 24 h and 21 days after injury, neuronal damage increased in the TBI and vehicle groups compared with the sham group (*P* < 0.01) (Figure 6(b)). Although neuronal damage was in the SC group higher than that the sham group at 24 h and 21 days after injury (*P* < 0.01), this variable was low in the SC group compared to the vehicle group at 24 h (*P* < 0.01) and 21 days (*P* < 0.05) after injury.

Figure 6(c) illustrates the results of microglia proliferation score in study groups at 24 h and 21 days after injury. At 24 h after injury, the score of microglia proliferation significantly increased in the TBI and vehicle groups compared with the sham group (*P* < 0.05). This increment was also observed at 21 days after injury in the TBI (*P* < 0.05) and vehicle (*P* < 0.01) groups compared with the sham group. As shown in Figure 6(c), microglia proliferation in the SC group decreases compared with the vehicle group at 24 h (*P* < 0.05) and 21 days (*P* < 0.01) after injury.

## 4. Discussion

Given the high mortality rate and life-long disabilities, lack of effective and definitive treatments [37], and suggestion of multipotential stem cell application due to heterogeneous nature of TBI [8], the effects of OMSCs on brain edema, anxiety-like behavior and long-term neurological, pathological, cognitive, and motor outcomes of diffuse TBI were investigated for the first time in the present research. It was shown the administration of OMSCs could improve neurological, cognitive, and motor functions, and anxiety-like behavior in TBI probably by decreasing brain edema, microglia proliferation, and neuronal damage. Therefore, the administration of OMSCs was successful in improving long-term outcomes of TBI. In the present study, an intravenous injection of OMSCs was used. Studies on using SC for TBI suggest that the best way of carrying SC to the injury site is IV injection, though the studies have shown that only 1% of the injected cells can reach the injury site in the brain [38–41].

Further studies are needed to find the appropriate time for SC injection. A study showed that injection of SC after 24 h of cerebral injury decreased the neuronal inflammation and increased the angiogenesis and neurogenesis [39]. In another study, exosome injection of MSCs after 15 minutes from the TBI could inhibit the inflammation by disturbing the injury cycle [42]. On the other hand, other studies stated that early administration of SC could not be effective due to the tissue inflammation, and delayed administration or repeating the dose may result in more effective outcomes [2, 43]. This study investigated repeating the injection of OMSCs in 1 and 24 h after the injury.

In agreement with our research, many studies have shown the increased cerebral water content after TBI [44, 45]. One of the main causes of developing cerebral edema is the increased inflammatory responses and the activation of inflammatory cascades that result in disruption of the BBB and further introduction of inflammatory agents and immune cells and consequently the development of cerebral edema [9]. In the present study, the increase in cerebral water content was prevented using OMSC. In a study, it was reported to be increased expression of tissue inhibitor of metalloproteinase 3 (TIMP3) by MSC, leading to a decrease in the permeability of the BBB in murine TBI model and thus improving the recovery after injury [46]. Therefore, the administration of OMSCs after TBI probably inhibited the development of brain edema partly by decreasing brain inflammation.

Motor dysfunctions are TBI common complications [47]. In the present study, the administration of OMSCs improved the motor impairments in the animals of the treatment group, so that the reduction in motor speed was resolved. Motor function improvement of the treatment group was also reported in the experimental study using NSCs for treating post-TBI cognitive and motor deficits [48]. Another study reported that embryonic stem cell administration after cerebral injury improved the motor function in mice [49]. The effectiveness mechanism of MSCs on posttraumatic motor outcomes is not completely understood. Function improvement following the administration of MSCs is thought to be caused by decreased inflammatory and oxidant factors and also neurogenesis stimulation [42]. MSCs may also improve motor function by inducing neurogenesis and axonal repair, improving tissue condition, and thus providing a better environment to advance tissue regeneration. It is albeit well demonstrated that administrating MSCs can significantly decrease posttraumatic inflammation [42], so that the animal motor function is improved; however, the molecular mechanism is unknown.

One of the important and common complications of TBI is also anxiety, which constitutes a significant part of the long-term symptoms and complications of TBI [50]. In the present study, posttraumatic anxiety-like behavior was indicated by a decrease in time spent in the open arms and a reduction in the entries into the open arms of EPM. Based on the studies and clinical trials done, TBI-induced anxiety and other psychological disturbances seem to be associated with the injury to the posterolateral area of the prefrontal lobe and the left side of basal nuclei. Progressive atrophy induced by injury in the area results in decreasing the recovery speed and defecting the cerebral function [51]. Administration of OMSCs increased the time spent in and the number of entries the open arms, indicating a reduction in anxiety in the treatment group.

In line of our results of memory, administration of mouse neural stem/progenitor cells (NSPCs) to mice with TBI could improve memory [52], whereas human NSPC, regardless of its positive efficacy and relieving motor deficits, had no effect on the development of long-term cognitive activity and memory [53]. In a study conducted by Zhou et al. (2019), it was shown that MSCs administration ameliorated neurological dysfunction and also memory and learning impairment after TBI [54]. Transplantation of MSCs overexpressing fibroblast growth factor 21 (FGF-21), as a neuroprotective protein, recovered hippocampal-dependent and independent learning and memory deficits in a mouse model of TBI [55]. In a study, it was shown that BMSCs transplantation could improve cognitive impairment through up-regulation of hippocampal GABAergic system in a rat model of chronic cerebral hypoperfusion [56].

In the present study, the neuronal damage and microglia proliferation were inhibited by the stem cell administration. Several studies demonstrated that MSCs could reduce inflammation following cerebral injury [30, 42, 43]. In a recent study, the administration of MSCs was found to significantly increase the *in vivo* and *in vitro* expression of IL-10 in the injured tissue. Treatment with MSCs also improved the motor skills and reduced the number of activated astrocytes and macrophages, thereby preventing the tissue from dying. MSCs can contribute to the regulation of dendritic cells, macrophages, and natural killer cells secreting cytokines [57]. They directly inhibit the proliferation of T cells and microglial cells and also decrease the secretion of inflammatory cytokines by dendritic cells, monocytes, and macrophages [58]. Moreover, MSCs can immigrate to the injured tissue [43]. They inhibit the proinflammatory cytokine activities and proliferation of T lymphocytes and microglial cells in the tissue. Also, MSCs increase the survival rate of the damaged cells by releasing the anti-inflammatory cytokines and immunologic regulatory factors [58]. The associated mechanism of action of stem cells is not fully known. It has been stated that after immigration to the injured tissues, these cells increase the immunologic tolerance in the environment and also the survival rate of the injured cells by inhibiting the release of proinflammatory cytokines [24]. More researvh is required for the confirmation.

## 5. Conclusion

The cerebral edema caused by TBI was reduced following OMSCs administration. Also, OMSCs administration decreased long-term motor, cognitive, and neurologic dysfunction and anxiety following TBI probably due to inhibiton of brain edema. Given the study results, we suggest that the use of OMSCs should be noted for prevention from long-term impairments of TBI and maybe other neurodegenerative disorders in future studies. Since the mechanism of action of MSC is not fully understood, given the abundant number of unanswered questions in this field, further studies are needed to understand the involved mechanisms and prove the safety of the method.

## Figures and Tables

**Table 1:** Neurological severity score.

	Point
Motor tests	
Raising rat by the tail (normal =0; maximum =3)	
Flexion of forelimb	1
Flexion of hind limb	1
Head moved >10° to vertical axis within 30 s	1
Placing rat on the floor (normal =0; maximum =3)	
Normal walk	0
Inability to walk straight	1
Circling toward the paretic side	2
Fall down to the paretic side	3
Sensory tests (normal =0; maximum =2)	
Placing test (visual and tactile test)	1
Proprioceptive test (deep sensation, pushing the paw against the table edge to stimulate limb muscles)	2
Beam balance tests (normal =0; maximum =6)	
Balances with steady posture	0
Grasps side of beam	1
Hugs the beam and one limb falls down from the beam	2
Hugs the beam and two limbs fall down from the beam, or spins on beam (>60 s)	3
Attempts to balance on the beam but falls off (>40 s)	4
Attempts to balance on the beam but falls off (>20 s)	5
Falls off: no attempt to balance or hang on to the beam (<20 s)	6
Reflexes absent and abnormal movements (normal =0; maximum =4)	
Pinna reflex (head shake when touching the auditory meatus)	1
Corneal reflex (eye blink when lightly touching the cornea with cotton)	1
Startle reflex (motor response to a brief noise from snapping a clipboard paper)	1
Seizures, myoclonus, myodystony	1
Maximum points	18

## Data Availability

The data of this study are available from the corresponding author upon reasonable request.

## Abbreviations

BDNF: Brain-derived neurotrophic factor
EPM: Elevated plus maze
FBS: Fetal bovine serum
FGF-21: Fibroblast growth factor 2
GDNF: Glia-derived neuroprotective factor
hADSCs: Human adipose-derived stem cells
H&E: Hematoxylin and eosin
ICP: Intracranial pressure
IGF-1: Insulin-like growth factor-1
L-DMEM: Low glucose Dulbecco's modified Eagle's medium
MWM: Morris water maze
MSCs: Mesenchymal stem cells
NSPCs: Neural stem/progenitor cells
NSS: Neurological severity score
NSCs: Neural stem cells
OAE: Open arm entries
OAT: Open arm time
OFT: Open field
OMSCs: Oral mucosal mesenchymal stem cells
PBS: Phosphate-buffered saline
SCs: Stem cells
TBI: Traumatic brain injury
TIMP3: Tissue inhibitor of metalloproteinase 3
VEGF: Vascular endothelial growth factor
Veh: Vehicle.

## References

[1] Guo S., Zhen Y., Wang A. (2017). Transplantation of bone mesenchymal stem cells promotes angiogenesis and improves neurological function after traumatic brain injury in mouse. *Neuropsychiatric Disease and Treatment*.

[2] Kim C., Park J.-M., Kong T. (2018). Double-injected human stem cells enhance rehabilitation in TBI mice via modulation of survival and inflammation. *Molecular Neurobiology*.

[3] Rossetti M. F., Cambiasso M. J., Holschbach M., Cabrera R. (2016). Oestrogens and progestagens: synthesis and action in the brain. *Journal of Neuroendocrinology*.

[4] Cheng G., Kong R. H., Zhang L. M., Kong R. H., Zhang L. M., Zhang J. N. (2012). Mitochondria in traumatic brain injury and mitochondrial-targeted multipotential therapeutic strategies. *British Journal of Pharmacology*.

[5] Das M., Mayilsamy K., Mohapatra S. S., Mohapatra S. (2019). Mesenchymal stem cell therapy for the treatment of traumatic brain injury: progress and prospects. *Reviews in the Neurosciences*.

[6] Jorge R. E., Robinson R. G., Moser D., Tateno A., Crespo-Facorro B., Arndt S. (2004). Major depression following traumatic brain injury. *Archives of General Psychiatry*.

[7] Maas A. I., Marmarou A., Murray G. D., Teasdale S. G. M., Steyerberg E. W. (2007). Prognosis and clinical trial design in traumatic brain injury: the IMPACT study. *Journal of Neurotrauma*.

[8] Dehghanian F., Soltani Z., Khaksari M. (2020). Can mesenchymal stem cells act multipotential in traumatic brain injury?. *Journal of Molecular Neuroscience*.

[9] Khaksari M., Soltani Z., Shahrokhi N. (2018). Effects of female sex steroids administration on pathophysiologic mechanisms in traumatic brain injury. *Translational Stroke Research*.

[10] Rabinowitz A. R., Levin H. S. (2014). Cognitive sequelae of traumatic brain injury. *The Psychiatric Clinics of North America*.

[11] Ayata C., Ropper A. H. (2002). Ischaemic brain oedema. *Journal of Clinical Neuroscience*.

[12] Soltani Z., Shahrokhi N., Karamouzian S. (2017). Does progesterone improve outcome in diffuse axonal injury?. *Brain Injury*.

[13] Sarkaki A. R., Khaksari Haddad M., Soltani Z., Shahrokhi N., Mahmoodi M. (2013). Time-and dose-dependent neuroprotective effects of sex steroid hormones on inflammatory cytokines after a traumatic brain injury. *Journal of Neurotrauma*.

[14] Meymandi M. S., Soltani Z., Sepehri G., Amiresmaili S., Farahani F., Aghtaei M. M. (2018). Effects of pregabalin on brain edema, neurologic and histologic outcomes in experimental traumatic brain injury. *Brain Research Bulletin*.

[15] Reis C., Gospodarev V., Reis H. (2017). Traumatic brain injury and stem cell: pathophysiology and update on recent treatment modalities. *Stem Cells International*.

[16] Cox C. S. (2018). Cellular therapy for traumatic neurological injury. *Pediatric Research*.

[17] Marynka-Kalmani K., Treves S., Yafee M. (2010). The lamina propria of adult human oral mucosa harbors a novel stem cell population. *Stem Cells*.

[18] Mizuno H., Tobita M., Uysal A. C. (2012). Concise review: adipose-derived stem cells as a novel tool for future regenerative medicine. *Stem Cells*.

[19] Sanchez-Ramos J., Song S., Cardozo-Pelaez F. (2000). Adult bone marrow stromal cells differentiate into neural cells in vitro. *Experimental Neurology*.

[20] Davies L. C., Locke M., Webb R. D. (2010). A multipotent neural crest-derived progenitor cell population is resident within the oral mucosa lamina propria. *Stem Cells and Development*.

[21] Torrente D., Avila M., Cabezas R. (2014). Paracrine factors of human mesenchymal stem cells increase wound closure and reduce reactive oxygen species production in a traumatic brain injury in vitro model. *Human & Experimental Toxicology*.

[22] Ganz J., Arie I., Ben-Zur T. (2014). Astrocyte-like cells derived from human oral mucosa stem cells provide neuroprotection in vitro and in vivo. *Stem Cells Translational Medicine*.

[23] Goodus M. T., Guzman A. M., Calderon F., Jiang Y., Levison S. W. (2015). Neural stem cells in the immature, but not the mature, subventricular zone respond robustly to traumatic brain injury. *Developmental Neuroscience*.

[24] Kota D. J., Prabhakara K. S., Toledano-Furman N. (2017). Prostaglandin E2 indicates therapeutic efficacy of mesenchymal stem cells in experimental traumatic brain injury. *Stem Cells*.

[25] Galindo L. T., Filippo T. R., Semedo P. (2011). Mesenchymal stem cell therapy modulates the inflammatory response in experimental traumatic brain injury. *Neurology Research International*.

[26] Chang C.-P., Chio C.-C., Cheong C.-U., Chao C. M., Cheng B. C., Lin M. T. (2013). Hypoxic preconditioning enhances the therapeutic potential of the secretome from cultured human mesenchymal stem cells in experimental traumatic brain injury. *Clinical Science*.

[27] Dehghanian F., Soltani Z., Farsinejad A., Jafari E., Bashiri H. (2020). *The effect of oral mucosal mesenchymal stem cells on long-term brain edema and lesion, anxiety-like behavior, and cognitive and motor outcomes in experimental traumatic brain injury*.

[28] Zhao Y., Gibb S. L., Zhao J. (2016). Wnt3a, a protein secreted by mesenchymal stem cells is neuroprotective and promotes neurocognitive recovery following traumatic brain injury. *Stem Cells*.

[29] Mahmood A., Lu D., Lu M., Chopp M. (2003). Treatment of traumatic brain injury in adult rats with intravenous administration of human bone marrow stromal cells. *Neurosurgery*.

[30] Zhang R., Liu Y., Yan K. (2013). Anti-inflammatory and immunomodulatory mechanisms of mesenchymal stem cell transplantation in experimental traumatic brain injury. *Journal of Neuroinflammation*.

[31] Soltani Z., Khaksari M., Shahrokhi N., Nakhaei N., Shaibani V. (2009). Effect of combined administration of estrogen and progesterone on brain edema and neurological outcome after traumatic brain injury in female rats. *Iranian Journal of Endocrinology and Metabolism*.

[32] Liu F., Chen M.-R., Liu J. (2016). Propofol administration improves neurological function associated with inhibition of pro-inflammatory cytokines in adult rats after traumatic brain injury. *Neuropeptides*.

[33] Bashiri H., Rezayof A., Sahebgharani M., Tavangar S. M., Zarrindast M. R. (2016). Modulatory effects of the basolateral amygdala *α*2-adrenoceptors on nicotine-induced anxiogenic-like behaviours of rats in the elevated plus maze. *Neuropharmacology*.

[34] Hamidkhaniha S., Bashiri H., Omidi A. (2019). Effect of pretreatment with intracerebroventricular injection of minocycline on morphine-induced memory impairment in passive avoidance test: role of P-CREB and c-Fos expression in the dorsal hippocampus and basolateral amygdala regions. *Clinical and Experimental Pharmacology and Physiology*.

[35] Sadeghinejad M., Soltani Z., Afzalpour M. E., Khaksari M., Pourranjbar M. (2019). What is the combined effect of intense intermittent exercise and Ginkgo biloba plant on the brain neurotrophic factors levels, and learning and memory in young rats?. *Pharmacological Reports*.

[36] Wee H.-Y., Lim S.-W., Chio C.-C., Niu K. C., Wang C. C., Kuo J. R. (2015). Hyperbaric oxygen effects on neuronal apoptosis associations in a traumatic brain injury rat model. *Journal of Surgical Research*.

[37] Gennai S., Monsel A., Hao Q. (2015). Cell-based therapy for traumatic brain injury. *British Journal of Anaesthesia*.

[38] Wiklander O. P., Nordin J. Z., O'Loughlin A. (2015). Extracellular vesicle in vivo biodistribution is determined by cell source, route of administration and targeting. *Journal of Extracellular Vesicles*.

[39] Zhang J., Huang X., Wang H. (2015). The challenges and promises of allogeneic mesenchymal stem cells for use as a cell-based therapy. *Stem Cell Research & Therapy*.

[40] Lin C.-S., Xin Z.-C., Dai J., Lue T. F. (2013). Commonly used mesenchymal stem cell markers and tracking labels: limitations and challenges. *Histology and Histopathology*.

[41] Qihao Z., Xigu C., Guanghui C., Weiwei Z. (2007). Spheroid formation and differentiation into hepatocyte-like cells of rat mesenchymal stem cell induced by co-culture with liver cells. *DNA and Cell Biology*.

[42] Ni H., Siaw-Debrah F., Hu J. (2019). Exosomes derived from Bone mesenchymal stem cells ameliorate early inflammatory responses following traumatic brain injury. *Frontiers in Neuroscience*.

[43] Toyoshima A., Yasuhara T., Kameda M. (2015). Intra-arterial transplantation of allogeneic mesenchymal stem cells mounts neuroprotective effects in a transient ischemic stroke model in rats: analyses of therapeutic time window and its mechanisms. *PloS One*.

[44] Khaksari M., Rajizadeh M. A., Bejeshk M. A. (2018). Does inhibition of angiotensin function cause neuroprotection in diffuse traumatic brain injury?. *Iranian Journal of Basic Medical Sciences*.

[45] Soltani N., Soltani Z., Khaksari M., Ebrahimi G., Hajmohammmadi M., Iranpour M. (2020). The changes of brain edema and neurological outcome, and the probable mechanisms in diffuse traumatic brain injury induced in rats with the history of exercise. *Cellular and Molecular Neurobiology*.

[46] Menge T., Zhao Y., Zhao J. (2012). Mesenchymal stem cells regulate blood-brain barrier integrity through TIMP3 release after traumatic brain injury. *Science Translational Medicine*.

[47] Levin H. S. (1995). Prediction of recovery from traumatic brain injury. *Journal of Neurotrauma*.

[48] Riess P., Zhang C., Saatman K. E. (2002). Transplanted neural stem cells survive, differentiate, and improve neurological motor function after experimental traumatic brain injury. *Neurosurgery*.

[49] Ikeda R., Kurokawa M. S., Chiba S. (2005). Transplantation of neural cells derived from retinoic acid-treated cynomolgus monkey embryonic stem cells successfully improved motor function of hemiplegic mice with experimental brain injury. *Neurobiology of Disease*.

[50] Herrmann N., Rapoport M. J., Rajaram R. D. (2009). Factor analysis of the Rivermead post-concussion symptoms questionnaire in mild-to-moderate traumatic brain injury patients. *The Journal of Neuropsychiatry and Clinical Neurosciences*.

[51] Fedoroff J. P., Starkstein S. E., Parikh R. M., Price T. R., Robinson R. G. (1991). Are depressive symptoms nonspecific in patients with acute stroke?. *The American Journal of Psychiatry*.

[52] Chrostek M. R., Fellows E. G., Guo W. L. (2019). Efficacy of cell-based therapies for traumatic brain Injuries. *Brain Sciences*.

[53] Harting M. T., Sloan L. E., Jimenez F., Baumgartner J., Cox C. S. (2009). Subacute neural stem cell therapy for traumatic brain injury. *Journal of Surgical Research*.

[54] Zhou Y., Shao A., Xu W., Wu H., Deng Y. (2019). Advance of stem cell treatment for traumatic brain injury. *Frontiers in Cellular Neuroscience*.

[55] Shahror R. A., Linares G. R., Wang Y. (2020). Transplantation of mesenchymal stem cells overexpressing fibroblast growth factor 21 facilitates cognitive recovery and enhances neurogenesis in a mouse model of traumatic brain injury. *Journal of Neurotrauma*.

[56] Long Q., Hei Y., Luo Q. (2015). BMSCs transplantation improves cognitive impairment via up-regulation of hippocampal GABAergic system in a rat model of chronic cerebral hypoperfusion. *Neuroscience*.

[57] Ooi Y. Y., Ramasamy R., Rahmat Z.'. (2010). Bone marrow-derived mesenchymal stem cells modulate BV2 microglia responses to lipopolysaccharide. *International Immunopharmacology*.

[58] Kremlev S. G., Palmer C. (2005). Interleukin-10 inhibits endotoxin-induced pro-inflammatory cytokines in microglial cell cultures. *Journal of Neuroimmunology*.

